# Correction: Syngas production by bi-reforming methane on an Ni–K-promoted catalyst using hydrotalcites and filamentous carbon as a support material

**DOI:** 10.1039/d0ra90072a

**Published:** 2020-06-26

**Authors:** Adelino F. Cunha, Sergio Morales-Torres, Luisa M. Pastrana-Martínez, António A. Martins, Teresa M. Mata, Nídia S. Caetano, José M. Loureiro

**Affiliations:** Laboratory of Separation and Reaction Engineering, Associate Laboratory LSRE-LCM, Department of Chemical Engineering, Faculty of Engineering, University of Porto Rua Dr. Roberto Frias 4200-465 Porto Portugal; LEPABE – Laboratory for Process Engineering, Environment, Biotechnology and Energy, Faculty of Engineering, University of Porto Rua Dr. Roberto Frias 4200-465 Porto Portugal; School of Engineering (ISEP), Polytechnic of Porto (P.Porto) R. Dr. António Bernardino de Almeida 431 4249-015 Porto Portugal afccu@isep.ipp.pt +351 22 83 21 159 +351 22 83 41 904; Carbon Materials Research Group, Department of Inorganic Chemistry, Faculty of Sciences, University of Granada Avenida de Fuentenueva, s/n 18071 Granada Spain semoto@ugr.es +34 958248526 +34 958240443

## Abstract

Correction for ‘Syngas production by bi-reforming methane on an Ni–K-promoted catalyst using hydrotalcites and filamentous carbon as a support material’ by Adelino F. Cunha *et al.*, *RSC Adv.*, 2020, **10**, 21158–21173, DOI: 10.1039/D0RA03264F.

The authors regret that the funding information was incorrectly shown in the acknowledgements section of the original manuscript. The corrected funding acknowledgement is as shown below.

This work was financially supported by: Base Funding – UIDB/00511/2020 of the Laboratory for Process Engineering, Environment, Biotechnology and Energy – LEPABE – funded by national funds through the FCT/MCTES (PIDDAC) and Base Funding – UIDB/50020/2020 of the Associate Laboratory LSRE-LCM – funded by national funds through FCT/MCTES (PIDDAC). This work was also financially supported by the Spanish Project ref. RTI 2018-099224-B100 from ERDF/Ministry of Science, Innovation and Universities – State Research Agency. Authors thank Prof. Alírio Rodrigues (LSRE) for supporting this research. AFC, AAM and TMM acknowledge the financial support from Fundação para a Ciência e a Tecnologia (FCT, Portugal) *via* research grants SFRH/BPD/105623/2015, SFRH/BPD/112003/2015, and IF/01093/2014, respectively. SMT and LMPM acknowledge the financial support from University of Granada (Reincorporación Plan Propio) and the Spanish Ministry of Economy and Competitiveness (MINECO) for a Ramon y Cajal research contract (RYC-2016-19347), respectively. Authors thank CONDEA Chemie (now SASOL) for supplying the sorbent PURAL MG30 impregnated with K_2_CO_3_ (aluminum magnesium hydroxide, 70% Al_2_O_3_).

The Royal Society of Chemistry apologises for these errors and any consequent inconvenience to authors and readers.

## Supplementary Material

